# The changing nature of worry about COVID-19 among the English public: a secondary analysis of 73 national, cross-sectional surveys, January 2020 to April 2022

**DOI:** 10.1136/bmjopen-2024-088027

**Published:** 2024-10-21

**Authors:** G James Rubin, Louise E Smith, Richard Amlôt, Nicola T Fear, Henry WW Potts, Susan Michie

**Affiliations:** 1 Institute of Psychiatry, Psychology and Neuroscience, King's College London, London, UK; 2 NIHR Health Protection Research Unit in Emergency Preparedness and Response, London, UK; 3 Behavioural Science and Insights Unit, UK Health Security Agency, London, UK; 4 King’s Centre for Military Health Research and Academic Department of Military Mental Health, King's College London, London, UK; 5 Institute of Health Informatics, University College London, London, UK; 6 Centre for Behaviour Change, University College London, London, UK

**Keywords:** COVID-19, Health Surveys, Public health, PUBLIC HEALTH

## Abstract

**Abstract:**

**Objectives:**

To investigate worry about COVID-19 during the pandemic, and whether worry was associated with phase of the pandemic, COVID-19 death and incidence rates, Government interventions (including lockdown and advertising), age, being clinically at-risk, ethnicity, thinking that the Government had put the right measures in place, perceived risk of COVID-19 to self and the UK, and perceived severity of COVID-19.

**Design:**

Secondary analysis of a series of cross-sectional surveys.

**Setting:**

73 online surveys conducted for the English Department of Health and Social Care between 28 January 2020 and 13 April 2022.

**Participants:**

Participants were people aged 16 years and over living in the UK (approximately 2000 per wave).

**Primary outcome measures:**

Our primary outcome was self-reported worry about COVID-19.

**Results:**

Rates of being ‘extremely’ or ‘very’ worried changed over time. Worry increased as infection rates increased and fell during lockdowns, but the association became less obvious over time. Respondents aged 60 years and over were less likely to be worried after the launch of the vaccination campaign, while those who were clinically at-risk or from a minoritised ethnic community were more likely to be worried. Higher worry was associated with higher perceived risk, and higher perceived severity of COVID-19. There was no evidence for an association with agreeing that the Government was putting the right measures in place to prevent the spread of COVID-19. The launch of graphic Government advertising campaigns about COVID-19 had no noticeable effect on levels of public worry.

**Conclusions:**

In future infectious disease outbreaks, spikes in worry may attenuate over time, although some sections of society may experience higher anxiety than others.

**STRENGTHS AND LIMITATIONS OF THIS STUDY**

- This analysis included data on self-reported worry from a large sample of participants, across more than 2 years of the COVID-19 pandemic.
- Participants were drawn from a panel interested in taking part in a range of surveys, reducing the risk that interest in the pandemic led to selection bias.
- The representativeness of the online panel in terms of attitudes to the pandemic is unknown.

## Introduction

 Outbreaks of emerging infectious diseases can cause high levels of anxiety and worry among the public. This can motivate people to take protective action,^1^ and influence economically relevant behaviours^2^ and mental health.^3^ High levels of worry and anxiety are not inevitable, and several factors have been proposed as influencing a population’s emotional response to an infectious disease outbreak. Understanding these factors can help those tasked with communicating with the public to understand how best to develop their messages, providing reassurance or motivation if required.

Timing presumably plays a role. There may be differences in how outbreaks that begin overseas are perceived when they are still geographically distant compared with when cases or deaths start to occur nearby.^4 5^ After an outbreak has reached a country, the first burst of activity by public health officials, politicians and the media may trigger greater public concern than similar activity that occurs after an outbreak has become established.^6 7^ The degree to which people show progressively smaller emotional responses to similar levels of infection risk over time (‘habituation’) is still uncertain.^8^

Second, the severity of an outbreak may influence responses. SARS, for example, triggered greater anxiety in the Hong Kong public than the subsequent H1N1 pandemic,^9^ while in the UK increasing levels of infection during the first wave of the H1N1 pandemic were associated with higher levels of worry.^6^ Evidence that fluctuations in the number of cases within a pandemic, and hence the level of risk, are associated with changing levels of concern suggest that people ‘adapt’ their risk perceptions to fit the changing context.^6 8^

Third, official responses can matter. Perceiving that the Government is failing to mount an adequate response to an outbreak can be concerning,^10 11^ while several studies have shown the potential for official statements or advertising campaigns to increase or decrease anxiety.^5 6^ In the UK, for example, concern has been raised over two advertising campaigns launched during the COVID-19 pandemic in which posters showed flame-red images of healthcare workers in personal protective equipment with messages such as ‘if you go out, you can spread it: people will die’^12^ and showing COVID-19 patients wearing oxygen masks with strap-lines such as ‘look him in the eyes and tell him the risk isn’t real’.^13^ Although the efficacy of fear appeals has been demonstrated in experimental studies,^14^ whether members of the public are influenced by such messages during real-world infectious disease outbreaks is a separate matter. It is plausible that even extensive, emotionally laden campaigns are drowned-out by the voluminous media reporting that accompanies pandemics and by people’s personal experiences.

Fourth, media attention probably matters. While media attention is often driven by Government messaging,^6^ the volume and tenor of media attention does not always align with official messaging.

Finally, individual differences may affect how people perceive outbreaks. For example, given the higher mortality rates for COVID-19 among older adults, those from minoritised ethnic groups and those with specific clinical conditions, it may be that people in these groups or who perceived themselves to be at risk were particularly worried by changes in the pandemic or in Government actions. It is unknown whether issues such as habituation, adaptation, or responses to advertising differ in different sections of society.

During the first 2 years of the COVID-19 pandemic, levels of worry in the UK population were tracked in 73 survey waves by the English Department of Health and Social Care (DHSC). Our team supported DHSC throughout the pandemic by proposing some items for their surveys and conducting detailed analyses of their data sets. In this paper, we used data from all survey waves to assess levels of worry and whether worry was associated with phase of the pandemic, COVID-19 death and incidence rates, Government interventions (including lockdown and advertising), age, being clinically at-risk, ethnicity, thinking that the Government had put the right measures in place, perceived risk of COVID-19 to self and the UK, and perceived severity of COVID-19.

## Method

### Design

A series of 73 anonymous cross-sectional surveys were conducted by BMG Research and then Savanta (both Market Research Society company partners) on behalf of the DHSC, and analysed by the COVID-19 Rapid Survey of Adherence to Interventions and Responses (CORSAIR) study. A detailed description of the methods and sampling strategy for the surveys is available elsewhere.^15^ Data collection began on 28 January 2020 and ended on 13 April 2022. Surveys were weekly for the first 5 months, then moved to fortnightly, with occasional exceptions.

We have previously published data from three survey waves that assessed worry during the first few weeks of the pandemic,^16^ and from five later waves to assess worry during the emergence of the Omicron variant.^17^

### Participants

Participants were eligible for DHSC’s surveys if aged 16 years or older and living in the UK. Participants completing a survey wave were unable to participate in the subsequent three waves. Participants had previously signed up to take part in online surveys and were recruited from two specialised research panel providers (Respondi, n=50 000; Savanta, n=31 500). Consent was implied by participants’ completion of the survey, as standard practice. Quotas (based on age and gender combined, and geographical region of the UK) were applied so that the sample was similar to the UK population for these characteristics. On completion, participants were reimbursed in points, which could be redeemed in cash, gift vouchers or charitable donations (up to 70 p per survey).

We selected participants living in England for this analysis, due to differing restrictions in the four UK nations.

### Study materials

#### Outcome measure

The surveys asked participants ‘overall, how worried are you about Coronavirus?’ on a 5-point scale from ‘extremely worried’ to ‘not at all worried’. Answers were recoded into a binary variable (‘extremely’ and ‘very’ worried vs ‘somewhat’, ‘not very’ and ‘not at all’ worried; ‘do not know’ coded as missing). Up until wave 5, the surveys referred to ‘Wuhan Coronavirus’ as this term had been used by some sections of the media in England.

### Explanatory variables

We used official sources for national and local incidence and death rates,^18^ focusing on 7 day rolling averages as these data informed Government communications to the public and were widely cited in the media.

The surveys asked participants to what extent they agreed that ‘the Government is putting the right measures in place to protect the UK public from Coronavirus (‘Wuhan Coronavirus’ up to wave 5)’ on a 5-point scale from ‘strongly agree’ to ‘strongly disagree’ (‘do not know’ coded as missing).

Participants were asked to what extent they thought COVID-19 (‘Wuhan Coronavirus’ up to wave 5) posed a risk to people in the UK and themselves personally on a 5-point scale from ‘major risk’ to ‘no risk at all’ (‘do not know’ coded as missing).

From wave 3, participants were asked to what extent they agreed that ‘Coronavirus would be a serious illness for me’ on a 5-point scale from ‘strongly agree’ to ‘strongly disagree’ (‘do not know’ coded as missing).

In all waves, participants were asked to report their gender, age, whether they had a dependent child in the household, whether they or a household member had a chronic illness, their employment status, the occupation of the highest earner in the household (from which socioeconomic grade can be derived) and ethnicity. Participants were also asked for their full postcode, from which index of multiple deprivation (2019) and region were derived. From wave 9, participants were asked how many people lived in their household. From wave 12, participants were asked what their first language was. From wave 14, participants were asked three items indicating to what extent in the past 7 days, they had been struggling to make ends meet, skipping meals they would usually have, and were finding their current living situation difficult on a 5-point scale from ‘strongly disagree’ to ‘strongly agree’. These were summed to give a financial hardship scale.

### Power

A sample size of 1700 allows a 95% CI of plus or minus 2% for the prevalence estimate for a survey item with a prevalence of around 50%. For associations, analyses using only two survey waves (n≈3400) would give us over 99.9% power to detect small differences (OR=1.68).^19^ Data from multiple survey waves were pooled, resulting in higher statistical power.

### Analysis

Data were analysed unweighted.

We graphically present rates of worry over the course of the pandemic in (1) the whole sample, (2) the sample split by age (60 years and over vs 59 years and younger), (3) the sample split by clinical at-risk status for COVID-19 (at risk vs not at risk)^20^ and (4) the sample split according to ethnicity (white British vs white other vs other minoritised ethnic group). We also plotted national incidence and death rates (7 day averages) and the launch dates of relevant Government interventions (including lockdowns and advertising campaigns). We used generalised estimating equations (GEEs) to investigate whether worry was univariably associated with timepoint in the pandemic (using survey wave as a proxy), age (60 years or older vs 59 years and younger), at-risk clinical status (not at-risk vs at-risk) or ethnicity (white British vs white other vs other minoritised ethnic group). We reran these analyses using seven groups (white British vs white other vs mixed vs Asian/Asian British vs Black/Black British vs Arab/other vs prefer not to say/do not know) to allow more detailed investigation of differences between minoritised ethnic groups. GEEs adjust for a proportion of participants completing multiple survey waves. Some participants were not included in these analyses as they were not able to be assigned unique participant identifiers (4.33%, n=5494).

To investigate associations between worry and perceptions that the Government had put the right measures in place, perceived risk of COVID-19 (to oneself and other people in the UK) and perceived severity of COVID-19, in the GEEs, we adjusted for personal and clinical characteristics (wave, region, gender, age (raw and quadratic term), having a dependent child in the household, being clinically at-risk, having a household member with a chronic illness, employment status, socioeconomic grade, index of multiple deprivation and ethnicity). An error in the questions about being clinically at-risk occurred in waves 31, 51, 52 and 53, which were excluded in analyses investigating being clinically at-risk. As questions about first language, number of people in the household and financial hardship were added later in the survey (from waves 12, 9 and 14, respectively), we conducted a sensitivity analysis adjusting for these variables (including waves 14–72 only).

### Patient and public involvement

Members of the public were not involved in this secondary analysis.

## Results

### Respondent characteristics

A total of 126 882 responses were included in analyses of worry (0.4% missing data due to answers of ‘do not know’, n=491/127 373). These responses were from at least 69 214 participants.

Of note, 53.3% of respondents were women (n=67 684; men 46.3%, n=58 792; prefer to self-describe 0.2%, n=302; prefer not to say 0.1%, n=104). Mean age was 48.3 years (SD=18.0, range 16 to over 100 years), with 31.1% being aged 60 years or over (n=39 468; 68.9% 59 years or younger, n=87 414). Respondents were slightly more likely to be white than the general population (82.8% white British, n=1 05 045; 6.2% white other, n=7835; 2.4% mixed, n=3059; 5.2% Asian/Asian British, n=6612; 2.3% Black/Black British, n=2964; 0.5% Arab/other, n=606 and 0.6% prefer not to say/do not know, n=761 (compared with 81.7% white in the 2021 census of England and Wales));^21^ 19.3% were clinically at-risk for COVID-19 (n=22 693/117 554; 80.7% not at risk, n=94 861).

### Variations in worry between January 2020 and April 2022

**Figure 1:**
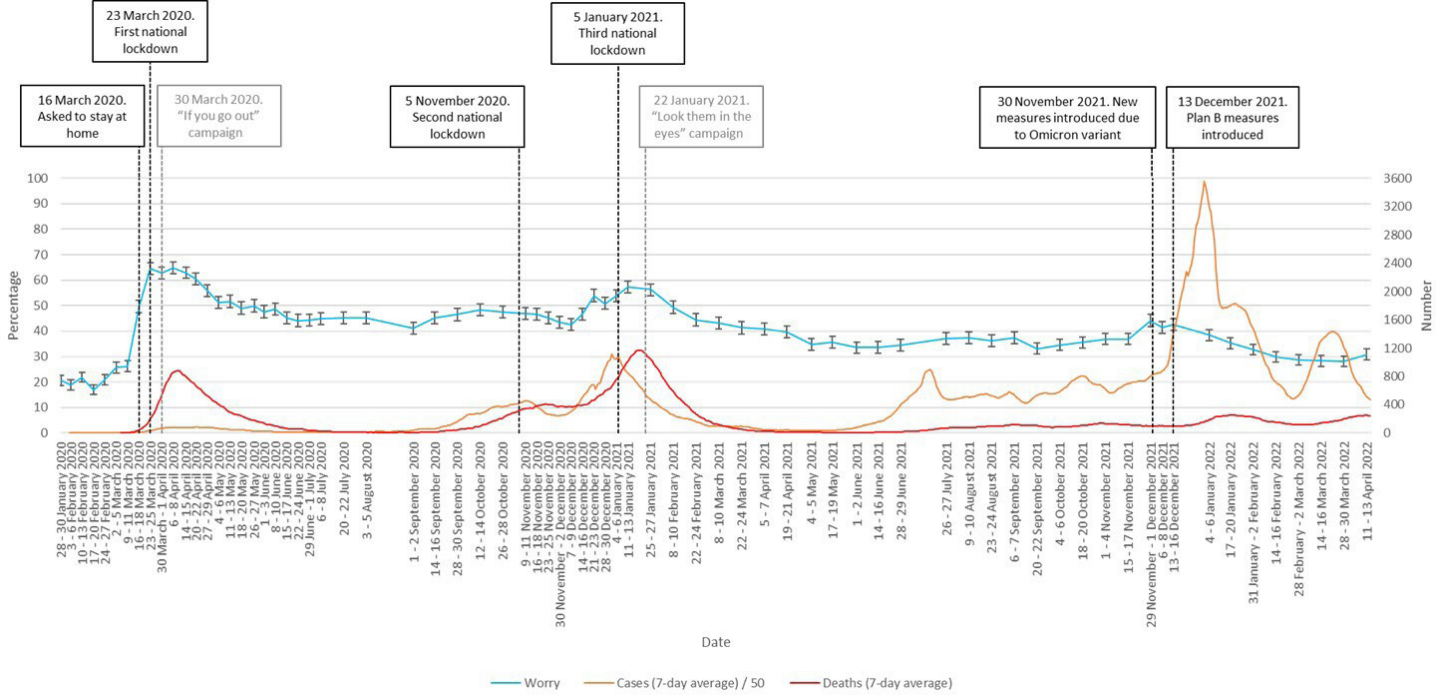
Percentage of people who were ‘very’ or ‘extremely’ worried between January 2020 and April 2022. Error bars are 95% CIs. Case numbers before June 2020 and in April 2022 are an underestimate as widespread testing was not implemented at this time. Black vertical lines denote major Government interventions. Grey vertical lines denote the launch of advertising campaigns.

There was significant variation in worry by survey wave over the course of the pandemic (χ^2^(72)=5541.3, p<0.001; see figure 1). Worry increased sharply in line with increasing cases at the start of the pandemic to a peak of 64.7% in April 2020, and dropped after the introduction of national restrictions. Worry increased again with the second wave of infections in winter 2020/2021, falling after the introduction of restrictions. There was only a slight increase in worry with the third wave of infections in winter 2021/2022. Aside from increases relating to these three events, there was a general trend for worry to decline over time, from 23 March 2020 onwards.

**Figure 2:**
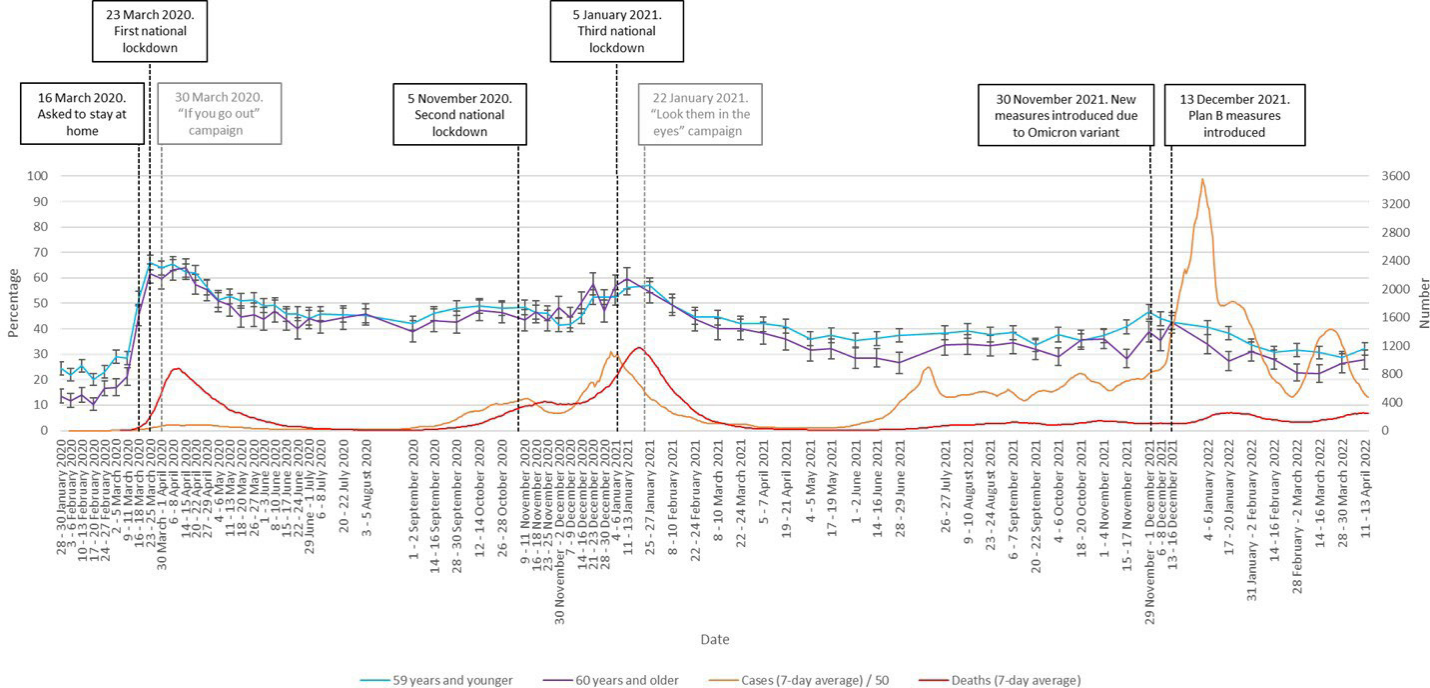
Percentage of people who were ‘very’ or ‘extremely’ worried between January 2020 and April 2022, by age (60 years and older vs 59 years and younger). Error bars are 95% CIs. Case numbers before June 2020 and in April 2022 are an underestimate as widespread testing was not implemented at this time. Black vertical lines denote major Government interventions. Grey vertical lines denote the launch of advertising campaigns.

Respondents aged 60 years and older were less likely to be very or extremely worried about COVID-19 (OR 0.87, 95% CI 0.84 to 0.90, p<0.001). Figure 2 shows that this difference started to manifest in spring 2021. This coincides with the rollout of the COVID-19 vaccination campaign in the UK, in which older adults were prioritised.

**Figure 3:**
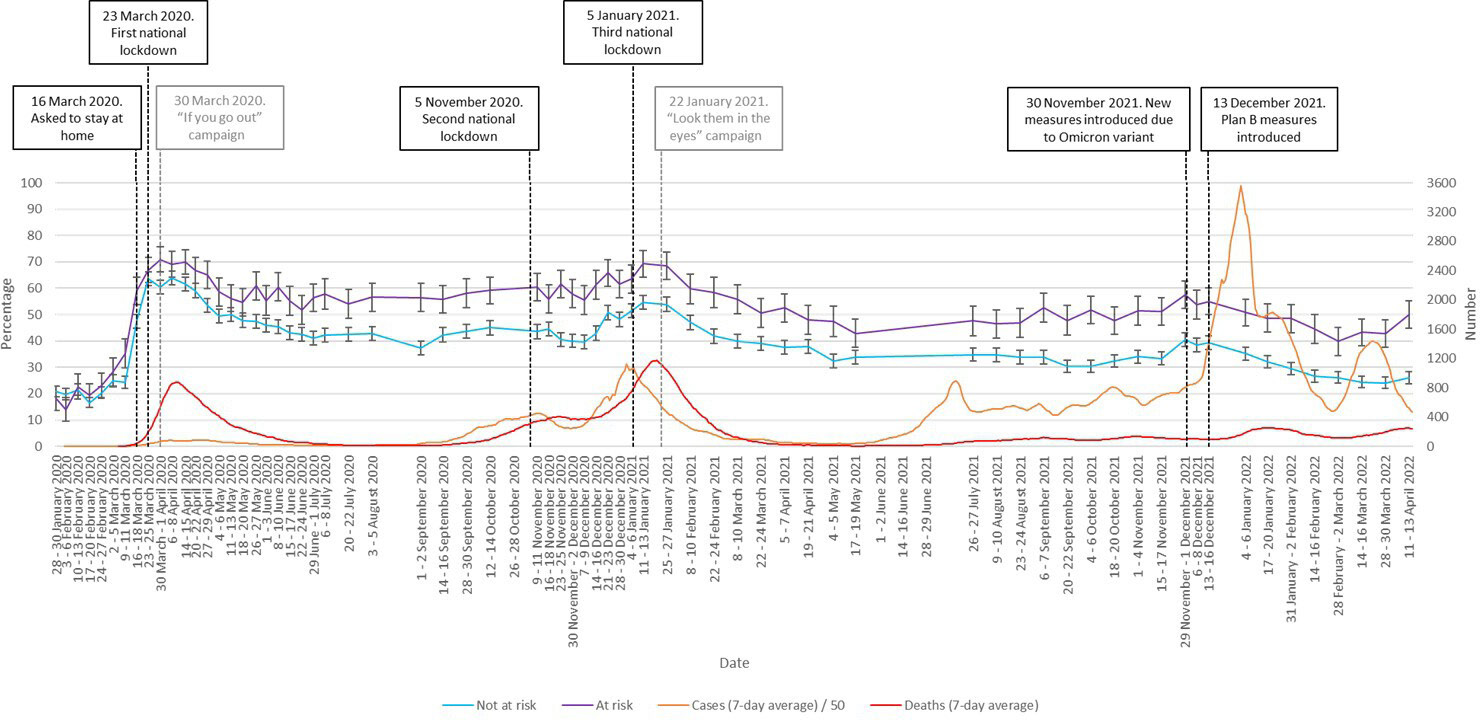
Percentage of people who were ‘very’ or ‘extremely’ worried between January 2020 and April 2022, by clinical at-risk status (at risk vs not at-risk). Error bars are 95% CIs. Case numbers before June 2020 and in April 2022 are an underestimate as widespread testing was not implemented at this time. Black vertical lines denote major Government interventions. Grey vertical lines denote the launch of advertising campaigns.

Respondents with an illness that puts them at-risk of COVID-19 were more likely to be very or extremely worried about COVID-19 (OR 1.77, 95% CI 1.71 to 1.83, p<0.001). This difference was seen from the start of the pandemic and throughout (figure 3).

**Figure 4:**
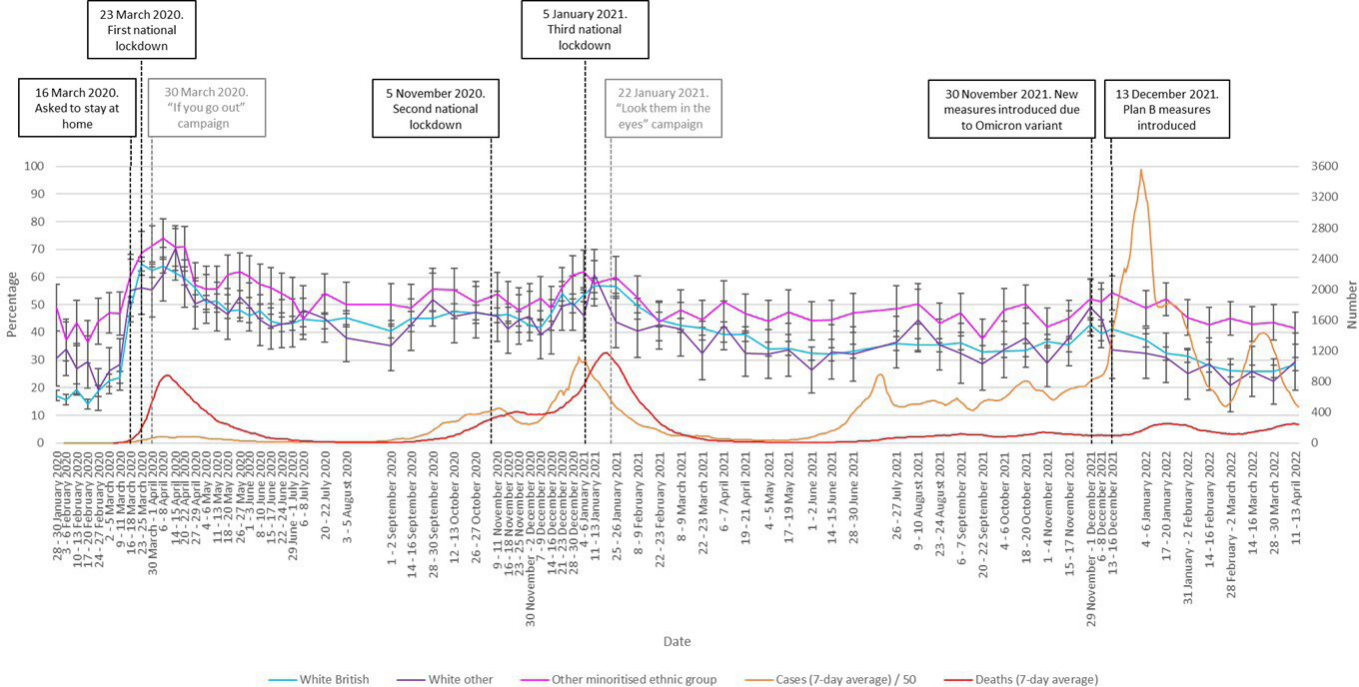
Percentage of people who were ‘very’ or ‘extremely’ worried between January 2020 and April 2022, by ethnicity (white British vs white other vs other minoritised ethnic group). Error bars are 95% CIs. Case numbers before June 2020 and in April 2022 are an underestimate as widespread testing was not implemented at this time. Black vertical lines denote major Government interventions. Grey vertical lines denote the launch of advertising campaigns.

People who identified as belonging to a minoritised ethnic group were more likely to be very or extremely worried about COVID-19 than those who identified as white British (OR 1.49, 95% CI 1.42 to 1.56, p<0.001; no evidence for a difference with white other: OR 1.03, 95% CI 0.97 to 1.09, p*=*0.31). This difference was observed throughout the pandemic (figure 4).

When looking at the non-white minoritised ethnic group in more detail, compared with white British, people identifying as mixed (OR 1.28, 95% CI 1.17 to 1.39, p<0.001), Asian/Asian British (OR 1.80, 95% CI 1.69 to 1.93, p<0.001), Black/Black British (OR 1.18, 95% CI 1.08 to 1.30, p<0.001) and Arab/other (OR 1.41, 95% CI 1.15 to 1.72, p*=*0.001) were more likely to be very or extremely worried.

### Associations with perceiving that the Government had put in place the right measures, and perceived risk

When adjusting for personal and clinical characteristics, there was no evidence for an association between worry and agreeing that the Government was putting the right measures in place to prevent the spread of COVID-19 (adjusted OR (aOR) 0.991, 95% CI 0.979 to 1.004, p=0.18). This remained the same when controlling for first language, living alone and financial hardship (aOR 0.993, 95% CI 0.980 to 1.006, p=0.30).

Greater worry about COVID-19 was associated with greater perceived risk of COVID-19 to people in the UK (aOR 3.41, 95% CI 3.34 to 3.48, p<0.001), greater perceived risk of COVID-19 to oneself (aOR 3.36, 95% CI 3.30 to 3.42, p<0.001) and greater perceived severity of COVID-19 (aOR 2.27, 95% CI 2.23 to 2.30, p<0.001). Results were similar when controlling for additional personal characteristics (perceived risk of COVID-19 to people in the UK, aOR 3.34, 95% CI 3.27 to 3.41, p<0.001; perceived risk of COVID-19 to oneself, aOR 3.30, 95% CI 3.23 to 3.37, p<0.001; perceived severity of COVID-19, aOR 2.26, 95% CI 2.22 to 2.30, p<0.001).

## Discussion

The pattern of worry identified in the data contains several distinctive features. Early news of the pandemic triggered limited worry in the English public. It was not until mid-March 2020 that levels of worry began to escalate. The initial low levels of the worry may have partly reflected the muted official response in the UK at this stage, low levels of trust in media reports about the spread of the outbreak^22 23^ and a perception that the outbreak was still geographically distant.^5^ Similar findings were seen in the UK during the swine influenza pandemic, with low levels of anxiety occurring in the earliest stages while the outbreak was still primarily restricted to Mexico.^6^

The escalation of worry in mid-March 2020 was the clearest and most dramatic change. This increase coincided with a series of changes in the UK at the time including worsening news from overseas, dramatic Government interventions and announcements, and an increase in deaths in the UK from three to over 100 between 10 and 26 March 2020, suggesting that the risk from the virus was neither ‘over hyped’ nor ‘over there’

While levels of worry appeared to climb as national case numbers increased throughout the pandemic, the strength of the association decreased over time. The strong associations we found between worry and perceived risk to self or others suggests that it may have been reductions in perceived risk that drove reductions in worry. Changes in the level of risk to members of the public occurred throughout the period, and the reduced emotional response probably reflected a rational adaptation to the changing context,^8^ at least in part. For example, the winter 2020/2021 rise in cases and the very large increase in cases caused by the Omicron variant was associated with less worry than in the first wave of infection. This was probably partly due to lower perceived and actual risk as a result of the COVID-19 vaccination campaign that began in December 2020 and the increasing number of people who believed that they had developed immunity after contracting COVID-19.^24^ This would also account for the faster decline in worry in older adults observed from December 2020, given that this group was prioritised to receive vaccination once it became available.

The reduced impact on worry of successive waves of infection might also have been caused by habituation among the public to the risk associated with COVID-19, something that has been observed before in relation to infectious and terrorism-related threats^6 25 26^ and has been reported elsewhere in relation to the COVID-19 pandemic.^8 27^ However, if any such habituation did occur, it was not apparent for members of the public who were in clinical ‘at risk’ groups during the winter 2020/2021 spike in infections. Worry within that group remained high throughout the first period of the pandemic, while during the December 2020 spike in infections worry in those who were clinically at risk returned to levels close to those seen in March 2020. If habituation to risk is a valid phenomenon, there appear to be important individual differences at play that determine who is affected by it.

The higher rates of worry among people from non-white minoritised ethnic communities that we observed, particularly among Asian and British Asian respondents, were apparent even in January 2020, suggesting that it reflected more generalised anticipation or concern about the likely impact of the pandemic for those communities. It is possible that this links to pre-existing and continuing low levels of trust in the Government among these communities, which affects the perception that the Government will take adequate steps to protect these communities during a crisis.^28^

The main impact of Government interventions was a reduction in worry following the imposition of the first and third national lockdowns and the reimposition of some public health and social measures during the Omicron wave. Conversely, we found no overall association between perceptions that the Government was putting the right measures in place to prevent the spread of COVID-19 and worry. Some caution may be required in interpreting this, as participant interpretations of the ‘measures’ being referred to may have changed over the pandemic and been influenced by media coverage. Perceptions that, for example, sufficient tests were being made available (a focus of the media in the early stages of the pandemic) may have had a different influence on worry compared with perceptions that the vaccination roll-out was being run effectively.

Although concerns have been expressed about the use of ‘fear-based messaging’ during the pandemic, we found no evidence that the initiation of the two media campaigns that are most commonly cited as problematic^29^ were associated with increased levels of worry. This suggests either that the public are more resilient to, or dismissive of, fear-based messaging than they are sometimes given credit for, that these specific adverts were not particularly worrying, or that in the context of a pandemic, the emotional tone of Government advertising is drowned out by the many of other sources of information that people are exposed to.

Our analysis has limitations. The data were generated from a series of cross-sectional online surveys with participants drawn from an existing panel of people interested in responding to surveys on a wide range of topics in return for compensation. The representativeness of such samples is not clear, although the fact that participants did not specifically volunteer because the survey related to the pandemic reduces the risk of bias related to interest in the topic.^30^ Several other data sets tracking variables similar to worry over time in the UK population^31^,^33^ found similar patterns despite using different measures and recruitment strategies, providing reassurance that the patterns in our data are representative.

The outcome measure was a single item, the reliability and validity of which are unclear. In terms of reliability, it may be that a multi-item scale would have provided a measure with greater reliability than a single item. In terms of validity, although the item specified ‘how worried are you’, we do not know whether responses were more affected by worry, or the related but separate concepts of fear or anxiety.

The observational nature of the data poses challenges for interpretation. Throughout the pandemic, changes in disease incidence, media attention, policy and public interest occurred in tandem. Teasing out the specific factors associated with changes in worry is therefore difficult.

Whether the trends observed in the COVID-19 pandemic in the UK will hold true in a future outbreak is difficult to predict. Some trends that we observed might be universal. For example, the tendency for a population to respond with low levels of concern to a risk that has not yet become established and for risk perceptions to reduce over time has been observed before. On the other hand, the highly disruptive nature of the COVID-19 pandemic and the subsequent debate as to whether policy responses were an under-reaction or over-reaction might have changed the way the public will respond to future infectious disease risks.

In terms of practical implications, our data suggest that in any future pandemic, it is likely that the initial spikes in population worry that will accompany the first infections or deaths within a country will wane over time. Given the importance of risk perception in driving behaviour change, this decline in worry may have implications for the maintenance of various behaviours that have health, social or economic significance. If it is correct that official advertising based on fear-appeals does little to affect this, then this suggests that public health officials who wish to encourage behaviour change should seek out other ways of doing so. With respect to research implications, we suggest that closer examination of the role of individual differences in determining habituation or adaptation to risk may be useful, given our finding that those most at risk from COVID-19 appeared to maintain high levels of worry throughout the initial months of the pandemic.

Overall, the data from the English population during the COVID-19 pandemic suggest that the spikes in worry that were triggered by increases in national incidence tended to decrease in magnitude as the pandemic progressed, and were reduced when the Government implemented interventions to reduce incidence rates. People from non-white minoritised ethnic communities or with medical risk factors tended to be more worried, while older adults prioritised for early vaccination saw their worry reduce once vaccination began. Advertising campaigns appeared to have no impact on population levels of worry.

## Data Availability

No data are available.
